# Exercise throughout Pregnancy Prevents Excessive Maternal Weight Gain during the COVID-19 Pandemic: A Randomized Clinical Trial

**DOI:** 10.3390/jcm11123392

**Published:** 2022-06-13

**Authors:** Cristina Silva-Jose, Miguel Sánchez-Polán, Rubén Barakat, Ángeles Díaz-Blanco, Vanessa Carrero Martínez, Fátima García Benasach, Irune Alzola, Michelle F. Mottola, Ignacio Refoyo

**Affiliations:** 1 AFIPE Research Group, Faculty of Physical Activity and Sport Sciences-INEF, Universidad Politécnica de Madrid, 28040 Madrid, Spain; cristina.silva.jose@upm.es (C.S.-J.); barakat.ruben@gmail.com (R.B.); 2 Gynecology and Obstetrics Department, Hospital Universitario Severo Ochoa de Leganés, 28911 Leganés, Spain; mariaangelesdiazblanco@gmail.com; 3 Gynecology and Obstetrics Department, Hospital Universitario Puerta de Hierro de Majadahonda, 28222 Majadahonda, Spain; vanessacarrerom@gmail.com (V.C.M.); fatimabenasach@hotmail.com (F.G.B.); 4 Clínica Zuatzu, 20018 Donostia-San Sebastián, Spain; ialzolae@gmail.com; 5 R. Samuel McLaughlin Foundation-Exercise and Pregnancy Lab, School of Kinesiology, Faculty of Health Sciences, Department of Anatomy & Cell Biology, Schulich School of Medicine & Dentistry, Children’s Health Research Institute, The University of Western Ontario London, London, ON N6A 3K7, Canada; mmottola@uwo.ca; 6 Sports Department, Faculty of Physical Activity and Sports Sciences-INEF, Universidad Politécnica de Madrid, 28040 Madrid, Spain; ignacio.refoyo@upm.es

**Keywords:** exercise, pregnancy, COVID-19 pandemic, weight gain

## Abstract

The purpose of this study was to examine the effects of a virtual exercise program throughout pregnancy during the COVID-19 pandemic on maternal weight gain. A randomized clinical trial (NCT NCT04563065) was performed. In total, 300 pregnant individuals were assessed for eligibility, and a total of 157 were randomized, of which 79 were in the control group (CG), and 78 were in the intervention group (IG). Those in the intervention group participated in a virtual supervised exercise program throughout pregnancy, 3 days per week. Fewer pregnant participants exceeded the weight gain recommendations in the IG group than in the CG (*n* = 4/5.9% vs. *n* = 31/43.1%, *p* = 0.001). Weight gain during pregnancy was lower in the IG than in the CG (9.96 ± 3.27 kg vs. 12.48 ± 4.87 kg, *p* = 0.001). Analysis of subgroups based on pre-pregnancy body mass index, showed significant differences in excessive maternal weight gain between study groups in normal-weight (IG, *n* = 0/0% vs. CG, *n* = 10/25%, *p* = 0.001) and those with overweight (IG, *n* = 2/18% vs. CG, *n* = 12/60%, *p* = 0.025). A virtual supervised exercise program throughout pregnancy could be a clinical tool to manage maternal weight gain during the COVID-19 pandemic by controlling excessive gain.

## 1. Introduction

Maternal weight gain and the metabolic intrauterine environment are important modifiable pregnancy epigenetic outcomes in relation to the future health of the mother and her child. Scientific evidence shows that failure to achieve appropriate maternal weight gain can influence other maternal, fetal, and newborn parameters [1]. Recently, it was shown that around 40% of pregnant individuals gain more weight than recommended. From a clinical point of view, excessive maternal weight gain is a common problem in various countries and has become one of the great challenges for health professionals responsible for health care during pregnancy [2].

The global epidemic of obesity is also a problem for health care providers because individuals starting pregnancy with a higher body mass index (BMI) are recommended to limit gestational weight gain. Individuals with higher BMI are more likely to have pregnancy complications and gain excessively which complicates pregnancy outcomes [3]. In addition, the impact of COVID-19 has generated a global crisis never before experienced, which affects physiological, emotional, mental, and social factors for all population groups, including those who are pregnant. Pregnant individuals are at the greatest risk for the resultant pandemic restriction effects (social isolation, greater stress to comply with the new health recommendations, etc.) [4].

The complications associated with confinement (no group support, reduced mobility, the distance between people, etc.), significantly affect the lifestyle of the pregnant individual and potentially remove one of the basic recommendations established by the international scientific community: a physically active lifestyle [5,6]. This threatens the achievement of recommended weight gain and is also a significant challenge for both pregnant individuals and healthcare professionals.

Exercise during pregnancy has been shown to be highly effective in controlling maternal weight gain [7]. However, the information available about this exercise/maternal weight gain relationship during the current pandemic is very scarce or null. This is due to the great difficulty in planning physical activity, especially through group programs, in the pregnant population who may or may not be vaccinated. Due to the COVID-19 pandemic restrictions, it is possible that this effective control of maternal weight gain during pregnancy has been affected compared with non-pandemic periods.

We investigated whether, during the current pandemic situation, a virtual supervised exercise program throughout pregnancy can be used to control excessive maternal weight gain for all BMI categories. Our study hypothesis is that exercise during pregnancy prevents excessive gestational weight gain during the pandemic period, resulting in a lower percentage of physically active individuals who gain excessive weight compared with those who are not as active.

## 2. Materials and Methods

### 2.1. Study Design

This study was developed with the collaboration between Hospital Universitario Severo Ochoa (Madrid), Hospital Universitario Puerta de Hierro (Madrid), Clínica Zuatzu (San Sebastián), and Universidad Politécnica de Madrid (UPM). A randomized clinical trial (registered in “ClinicalTrials.gov”, registration number: NCT04563065) was performed, and the study protocol was approved by the Ethical Committee at UPM; this is a secondary analysis (two articles were previously published with the same registration number but different aims) [8,9]. Pregnant individuals were randomly assigned to an intervention (IG) or control group (CG). The clinical records from the hospitals were used to collect personal and medical data from the participants.

### 2.2. Patient and Public Involvement

Patients were included at the time they attended their obstetric consultation and received approval from their healthcare provider to perform physical exercise. The research question is a priority, based on the alarming levels of obesity in general around the world and especially in this population stratum, studied in detail in recent years. To this is added the current situation of the ongoing pandemic, which could aggravate and facilitate the increase in sedentary behaviors, assuming a multitude of complications for both mothers, fetuses, and newborns. Thus, the clinical trial is oriented based on the physical and emotional needs of the evolution of the pregnancy, and the intervention is structured according to international reference guidelines and recommendations.

During the trial, the women were aware of the type of intervention they were undergoing, sharing their diverse views and feelings that the study reported as a benefit to them. Lastly, the pregnant women had resources and guidelines at their disposal during their active participation in the study, and once the results are obtained, a document will be made available to the women with the main findings.

### 2.3. Participants and Randomization

**Figure 1:**
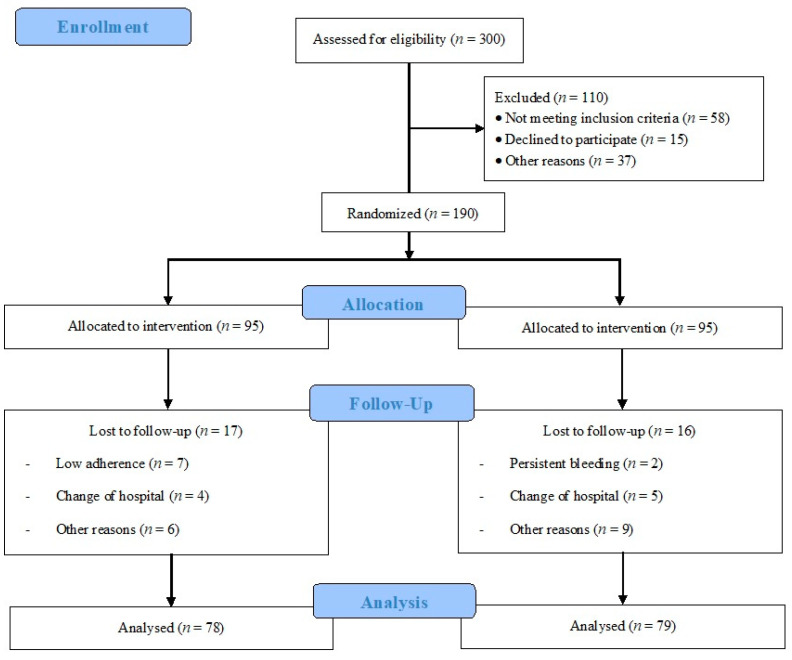
Study population flowchart.

A total of three hundred Spanish-speaking (Caucasian) pregnant individuals from hospital obstetric records (Figure 1) were assessed for eligibility. Inclusion/exclusion criteria were determined at this initial visit by the attending obstetrician. Individuals with singleton and uncomplicated pregnancies, with no history or risk of preterm delivery and not participating in any other trial or exercise program, were invited to participate. Those not planning to give birth in the reference hospitals and not under medical follow-up throughout pregnancy were not included in the study, nor were individuals having any serious medical conditions (contraindication) that prevented them from exercising safely [10,11,12].

Randomization was developed by an informatic software generating a list of random number blocks sequence by one researcher (assessing allocation 1:1 to each group). This sequence was blinded to the other researchers, health providers, and participants. The randomization process (sequence generation, allocation concealment, and implementation) was conducted by three different researchers from those who performed the physical activity program and supervision.

Firstly, women attended prenatal care consultations, in which obstetricians and gynecologists excluded those who had obstetrical contraindications to exercise. Then, women were informed about the development of the study, and after their signed informed consent, they were randomized with the randomization process.

After individuals provided written informed consent, 190 gravidae over 18 years who did not have any type of medical contraindication for physical exercise and were receiving normal obstetric care were randomized to either an exercise intervention (IG; *n* = 95) or usual care (control, CG; *n* = 95) group.

### 2.4. Intervention

A virtually supervised exercise program between 8–10 and 38–39 weeks of pregnancy was performed by the intervention group (IG). An average of 80–85 training sessions conducted by a qualified exercise specialist was originally planned for each participant in the event of no preterm delivery, and a minimum of 80% adherence to the exercise program was required to be included in the analysis of the results.

The virtually supervised exercise program involved three weekly sessions of 55–60 min of varied activities following an established model divided into the following seven parts [13]:

1. Warm-up with general exercises: range of motion varied, but impact activities were not included (avoiding jumps and falls);
2. Aerobic exercises: exercises were performed to increase the intensity up to that of moderate activities, 55–65% of heart rate reserve using the Karvonen formula and a range of 12–14 of Borg Rate of Perceived Exertion Scale (somewhat hard);
3. Muscle strengthening and general toning exercises of the whole body: Exercises for the lower extremities (calf, quadriceps, hamstrings, adductors, and abductors) and torso (abdominal, pectoral, shoulders, and paravertebral musculature) were included. The muscle groups to train must be distributed into the three weekly sessions. During each session, one or two sets of 10–12 repetitions must be performed from each muscle group using barbells (2–3 kg/exercise) or low-to-medium resistance (elastic) bands (TheraBand’s). Exercises for the most weakened muscle groups (overall regarding lower limbs and thoracolumbar muscles) during pregnancy were also included, as the aim was to avoid muscular decompensation;
4. Coordination and balance exercises: simple eye–hand and eye–foot coordination tasks were performed with sports equipment, as well as body axis balance exercises;
5. Strengthening the pelvic floor muscles: Kegel exercises were performed;
6. Cool-down section of 7–8 min: the aim was to gradually lower the intensity of work with flexibility, stretching, and relaxation exercises;
7. Final discussion: The aim of this section was to have the pregnant participants express clearly and openly the sensations and perceptions they experienced during the training session. This part was conducted only during the group virtual session.

The exercise program was provided by two modalities:

- Individual work (two weekly sessions): recorded sessions, with complete visual information and instructions regarding the exercises to be performed. These sessions were designed so that pregnant participants could follow easily and intuitively, with simple access for downloading;
- Group work (one weekly session): classes were supervised online through the video platform (Zoom Video Communications Inc., San José, CA, USA).

Control of attendance at the sessions was as follows:

- Participants had to send the two sessions weekly by means of videos to one of the researchers as evidence of the exercise session performed;
- Attendance was easily monitored based on the registration tool obtained by the computer application.

### 2.5. Usual Care (Control) Group

Those randomly assigned to the CG received general advice from their health care provider (obstetrician and midwife) including positive effects of physical activity or nutritional recommendations. Participants in the CG had their usual visits with health care providers during pregnancy, which were equal to the exercise group. Individuals in the CG were asked about their exercise once each trimester, using a “decision algorithm” (via telephone) [14], in which an assessment of the level of physical activity weekly was performed (setting up the limit in 150 min of physical activity weekly, following international clinical guidelines of physical activity during pregnancy) [10,12].

### 2.6. Outcomes

#### 2.6.1. Primary Outcome

Data corresponding to maternal weight gain and initial BMI were obtained from obstetric visits (hospital records). Gestational weight gain was calculated on the basis of pre-gravid weight and weight at the last clinic visit before delivery.

BMI was calculated as weight (kg) divided by height (m)^2^, and individuals were classified as underweight (BMI < 18.5 kg/m^2^), normal weight (BMI ≥ 18.5 to 24.9 kg/m^2^), overweight (BMI ≥ 25 to 29.9 kg/m^2^), and obese (BMI ≥ 30 kg/m^2^).

Gestational weight gain was classified according to the 2009 Institute of Medicine (IOM) guidelines [13]. Excessive body weight gain was determined for pre-pregnancy BMI categories for each participant; >18 kg for underweight; >16 kg for normal; >11.5 kg for overweight; and >9 kg for obese [15].

#### 2.6.2. Secondary Outcomes

Maternal gestational age, type and mode of delivery, birth weight, birth length, Apgar Scores, and pH of the umbilical cord blood were collected from hospital perinatal records.

Demographic and personal data were obtained at the first prenatal visit and hospital records. All data were provided by the SELENE (Community of Madrid, Spain) platform that manages all hospital obstetric records.

### 2.7. Statistical Analysis

Version 25.0 of IBM SPSS for Windows (IBM Corporation, Armonk, NY, USA) was used. Preliminary assessments were conducted using the Kolmogorov–Smirnov test to screen for violations of normality. Pearson’s chi-square test was used to compare the obtained frequencies of maternal BMI, smoking previous and during, previous miscarriages, parity, and employment occupation between the IG and CG. In addition, this same test was used to determine excessive weight gain relating to the group (overall and based on pre-pregnancy BMI).

Independent *t*-tests were used to assess the differences in age, gestational age, weight, and height between the intervention and control groups. This was also used to assess the differences between groups (overall and based on pre-pregnancy BMI) of gestational weight gain and weights at week 22 + 2, 28 + 1, 36 + 1, and final recorded weight.

Multiple linear regression was used to examine maternal weight gain as a function of maternal characteristics (initial BMI, age, occupation, smoking during pregnancy, parity, and previous miscarriage) and study groups. Adjusted R^2^ was used to explain the proportion of the total variance of the variable explained by the regression.

One-way ANOVAs were performed to examine birth weight differences related to maternal stratified gestational weight gain between groups. The effect size was obtained using the partial index η^2^.

Two-factor repeated-measure ANOVA was used to assess changes in gestational weight throughout pregnancy within the IG and CG and between both groups. Multiple comparisons were made using the Bonferroni test. The effect size was obtained using the partial index η^2^.

Data for continuous variables are presented as means and standard deviations, and those of the nominal variables are presented as frequencies and percentages. The level of statistical significance was set at *p* < 0.05.

### 2.8. Sample Size Calculation

Power calculations for the primary outcome (excessive maternal weight gain) were based on previous studies [16] and used a prevalence of ~10% in the intervention group and 30% in the usual care group. Under these assumptions, a two-sample comparison (χ^2^) with a 5% level of significance and statistical power of 0.90 yielded a study population of 82 participants in each group. Assuming a maximum loss to follow-up of 15%, we decided to recruit 95 participants for each of the study groups [17,18].

## 3. Results

A total of 300 women (from 26 September 2020 to 30 June 2021) were assessed for eligibility, and 110 were not included: Among them, 58 did not meet the inclusion criteria, 15 declined to participate, and 37 for other reasons. Participants were divided into IG (*n* = 95) and CG (*n* = 95). In the IG, 20 women were lost to follow-up, 8 had low adherence, 5 changed hospitals, and 7 for other reasons. In the CG, 16 women were lost to follow-up: 2 had persistent bleeding, 5 changed hospitals, and 9 for other reasons (6 of them for not being within the parameters of the decision algorithm). Therefore, 75 individuals in the IG and 79 in the CG were analyzed (Figure 1). Finally, for the analysis of gestational weight gain, data from 68 women in the IG and 72 in the CG were used, as the data were not registered according to the protocol in the hospital.

Table 1 shows the characteristics at baseline of the pregnant participants. No significant differences (*p* > 0.05) in maternal characteristics were found between the groups.

After performing a multiple linear regression, the weight gain prediction model (y = 7.962 + 2.233x) was dependent on belonging to the IG or CG (F _(1,122)_ = 8.897; *p* = 0.003; R2 = 0.060). Maternal characteristics—namely, initial BMI (*p* = 0.353), age (*p* = 0.362), occupation (*p* = 0.111), smoking during pregnancy (*p* = 0.361), parity (*p* = 0.264), and previous miscarriage (*p* = 0.449)—were excluded from the model.

Pearson’s chi-square test showed significant differences in EGWG between the IG (*n* = 4, 5.9%) and CG (*n* = 31, 43.1%), (χ^2^ (28) = 25.77; *p* < 0.001), with the CG showing a higher percentage of EGWG.

Higher percentages of EGWG were found in the CG in normal weight (χ^2^ (11) = 13.80; *p* < 0.001) and overweight subgroups (χ^2^ (3) = 5.01; *p* = 0.025). In the normal weight subgroup, 25% of the CG (10 of 40) experienced EGWG, while in the IG, there were none. In addition, 60% of participants overweight in the CG (*n* = 12) had EGWG, while in the IG, this number was 18% (*n* = 2) (Table 2).

Significant differences were found in total gestational weight gain between groups, IG (9.96 ± 3.27) vs. CG (12.48 ± 4.87 kg), (*p* < 0.001) shown in Table 3.

Performing an intergroup analysis, differences were found between groups (F _(1,132)_ = 8.155; *p* = 0.005; η^2^ = 0.050). When stratified by pre-pregnancy BMI, significantly higher gestational weight gains were found in the CG subgroup of normal weight women (IG = 10.27 ± 3.29 vs. CG 12.14 ± 4.75; *p* = 0.032) and overweight women (IG = 9.20 ± 3.11). vs. (CG 12.90 ± 5.04; *p* = 0.036). However, no significant intergroup differences were found in the interaction between the group and BMI categories (F _(3,132)_ = 0.544; *p* = 0.544; η^2^ = 0.012), nor in the comparison within the BMI subgroup (F _(1,132)_ = 0.557; *p* = 0.644; η^2^ = 0.013).

In general, the results showed that weight was significantly higher in the control group at week 28 + 4 days, week 36 + 1 day, and at the end of pregnancy 39 + 5 days (*p* < 0.05) (Table 4).

Using the repeated two-factor ANOVA, no significant intergroup differences were found (IG vs. CG) in weight (F _(4,307)_ = 2.353; *p* = 0.080; η^2^ = 0.0620) during the whole process of pregnancy. Similarly, no significant differences were found between groups based on pre-pregnancy BMI (F _(12,307)_ = 0.834; *p* = 0.573; η^2^ = 0.021).

After performing an intragroup analysis, significant differences were observed within the IG in the weight at different moments of pregnancy (F _(4.92)_ = 78.64; *p* < 0.001; η^2^ = 0.584). Multiple comparisons showed significantly higher weight in the final weight (+9.04 kg), in the 36 + 1 week (+8.5 kg), in the 28 + 4 week (+6.01 kg), and in the 22 + 2 week (+1.69 kg), compared with initial weight (*p* < 0.001). Furthermore, weights at final week (+7.35 kg), at 36 + 1 week (+6.81 kg), and 28 + 4 week (+4.32 kg) were significantly higher than weight at 22 + 2 week (*p* < 0.001). However, no differences were found in the rest of the comparisons made (Table 4).

On the other hand, within the CG, no significant differences were found in weight gain (F _(4,153)_ = 206.48; *p* < 0.001; η^2^ = 0.755). The distribution of weight increase in the CG was greater in in the final week (+11.85 kg), in the 36 + 1 week (11.82 kg), and in the 28 + 4 week (+8.34 kg), compared with the initial weight (*p* < 0.001). Additionally, weights at final week (+10.15 kg), at 36 + 1 week (+9.58 kg), and 28 + 4 week (+6.67 kg) were significantly greater than weight at week 22 + 2 (*p* < 0.001). Lastly, the weight in the final week (+3.48 kg) and in week 36 + 1 (+2.91 kg) were higher than weight at week 28 + 4 (*p* < 0.001).

Considering the secondary variables (Table 5), significant differences were only found in the mode of delivery (χ^2^ (6) = 5.97; *p* < 0.049), with more instrumental and c-sections in the CG.

## 4. Discussion

The objective of this study was to examine the effects of a virtual supervised exercise program throughout pregnancy during the COVID-19 pandemic. The main novelty of our study was to provide pregnant individuals with an easily accessible tool (virtual exercise) in order to combat the detrimental effects of COVID-19 and its associated complications (reduced mobility, no grouping activities). Due to the impossibility of face-to-face sessions, our intervention was based on the transfer to a virtual mode of an already scientifically successful program [8,13] consisting of various activities such as light resistance, toning, aerobic dance, balance-coordination exercises, pelvic floor training, and easily including a final talk. The high adherence rate achieved demonstrates the satisfaction of all BMI categories, as well as the urgent need to incorporate exercise into obstetric protocols as a fundamental alternative to the current and future situation caused by the COVID-19 pandemic. Additionally, those women who achieved a low adherence rate alleged that due to their labor journeys they could not perform all the weekly sessions. To our knowledge, this is currently the only RCT examining the influence of a virtual supervised exercise program on maternal weight gain throughout pregnancy during the restricted situation caused by COVID-19.

It can be seen that, in the baseline characteristics, there were some differences (without being statistically significant) between the intervention and control groups regarding parity, occupation, BMI, previous miscarriages, and smoking previously pregnancy, but the robust randomization process and blinding of the sequence allowed us to determine that these differences were casual, and with a large sample size, they would not be shown.

Overall, the main finding of the present study was that a virtual, exercise-based intervention performed by healthy pregnant women three times per week from 12–14 weeks of gestation until 38–39 weeks was successful at both preventing excessive gestational weight gain based on the IOM 2009 guidelines and reducing mean gestational weight gain. BMI subgroup analysis showed significant differences in normal weight and overweight categories for both excessive maternal weight gain and total maternal weight gain, with higher values observed in the CG. No differences were found in the underweight or obese categories between the groups, which could be due to the small sampling size.

One of the most relevant results that rigorously showed the impact of the exercise program throughout pregnancy was the weight gain accumulated by the pregnant individuals in each trimester (Table 4). Greater progressive maternal weight gain was observed in the CG throughout pregnancy, which showed better control of weight gain in the IG, especially during the second half of pregnancy (week 28 + 4 days to final weight). Prior to the pandemic situation, other experimental studies have examined the effects of gestational exercise on maternal weight gain and found positive effects of programmed exercise on weight gain control [16,19,20,21] even in the recovery of pre-pregnancy weight [22]. Review studies have also examined this issue, reporting better control of maternal weight gain in the exercise groups [23,24,25,26].

Although there were no statistically significant differences between groups in the obese BMI category (>30), this could be explained because of the small sampling size, but it seems that physical activity intervention could be beneficial for obese women. It is necessary to develop an intervention with a large sampling size to demonstrate this effect.

To our knowledge, this is the first study during the COVID-19 pandemic to use programmed virtual exercise during pregnancy as a preventive tool in the management of maternal weight gain. Our results showed relevant effectiveness in the safe control of excessive weight gain, which is highly applicable clinically, given the worrisome situation generated by new increases in virus infections and associated complications to the pregnant population.

The capacity of supervised exercise as a promoter of maternal, fetal, and newborn well-being (this time given virtually) is again shown to confirm the need to recommend a healthy lifestyle, including exercise throughout pregnancy, by health practitioners (obstetricians and midwives) responsible for gestational wellness. This is reflected in the main strength of the present study to overcome the limitations imposed by confinement resulting from the pandemic (reduced mobility, no grouping), through a virtual, safe, and attractive exercise program with high acceptance by pregnant participants, as demonstrated by the adherence achieved (>80%).

The first limitation of our study was that we did not assess nutrition or energy intake, which, in the current pandemic situation, means a potential risk bias; however, all women had standard care and information regarding a healthy lifestyle during pregnancy by health practitioners (obstetricians and midwives). Additionally, the non-stratification of the randomization process was a limitation that will be resolved in our future studies. In future research lines, we would like to perform a larger sampling size study to demonstrate more differences between physically active women and sedentary ones, and develop other advanced statistical analyses with pre-pregnancy BMI levels, occupation, and physical activity before the gestation.

## 5. Conclusions

The results of the present study indicated that a virtual supervised exercise program throughout pregnancy may act as a clinical tool in the management of maternal weight gain during the COVID-19 pandemic, controlling excessive weight gain. This is especially important because of the high risks that the pandemic situation generates regarding movement restrictions and associated complications for the mother, fetus, and newborn.

## Figures and Tables

**Table 1:** Maternal characteristics.

Maternal Characteristics			
Variable	Intervention Group (*n* = 75)	Control Group (*n* = 79)	
**Age (years)**	33.85 ± 4.05	33.49 ± 5.37	
**Maternal height (m)**	1.63 ± 0.06	1.62 ± 0.06	
**Maternal weight (kg)**	64.02 ± 16.92	66.99 ± 15.42	
**BMI (*n*/%)**	23.47 ± 6.37	25.01 ± 4.54	
<18.5	5/6.7	4/5.1	
18.5 to 24.9	54/72.0	43/54.4	
25 to 29.9	11/14.7	22/27.8	
>30	5/6.7	10/12.7	
**Parity** † **(*n*/%)**			
None	54/72.0	45/56.9	
One	17/22.7	26/32.9	
Two or more	4/5.3	8/10.2	
**Smoking previous pregnancy**			
No	57/76.0	50/63.3	
Yes	18/24	29/36.7	
**Occupation (*n*/%)**			
Active job	35/46.7	27/34.2	
Sedentary job	33/44.0	36/45.6	
Homemaker	7/9.3	16/20.2	
**Previous miscarriage (*n*/%)**			
None	56/74.7	48/60.8	
One	16/21.3	22/27.8	
Two or more	3/4.0	9/11.4	

Data are expressed as mean ± SD unless otherwise indicated.

**Table 2:** Cases of excessive weight gain measured at delivery in both study groups divided by body mass index (BMI): IG—intervention group; CG—control group.

	IG (*n* = 68)		CG (*n* = 72)		*p*-Value
Pre-Pregnancy BMI	*n*	*n*/%	*n*	*n*/%	
<18.5	4	0/0	3	1/33.3	0.212
18.5–24.9	49	0/0	40	10/25	**0.001**
25–29.9	11	2/18	20	12/60	**0.025**
>30	4	2/50	9	8/88.9	0.125

**Table 3:** Total weight gain at delivery in kg by BMI subgroups.

	IG (*n* = 68)		CG (*n* = 72)		*p*-Value
Total Weight Gain kg	mean ± SD		mean ± SD		
	9.96 ± 3.27		12.48 ± 4.87		0.001
Pre-pregnancy BMI (kg/m^2^)	*n*	mean ± SD	*n*	mean ± SD	
<18.5	4	11.42 ± 2.12	3	14.25 ± 7.40	0.487
18.5–24.9	49	10.27 ± 3.29	40	12.14 ± 4.75	**0.032**
25–29.9	11	9.20 ± 3.11	20	12.90 ± 5.04	**0.036**
>30.0	4	8.5 ± 3.33	9	12.41 ± 4.87	0.136

**Table 4:** Gestational mean weight (kg) and gestational weight gain (kg) in study groups for each measured time throughout pregnancy: IG—intervention group; CG—control group.

	IG (*n* = 68)		CG (*n* = 72)		*p* Value
	Mean ± SD	Mean ± SD	Mean ± SD	Mean ± SD	
Pre-pregnancy weight (kg)	64.02 ± 16.92	-	66.99 ± 15.42	-	0.258
Gestational Week 22 + 2	65.71 ± 12.78	1.69 ± 0.41	68.66 ± 13.52	1.67 ± 0.79	0.178
Week 28 + 4 days	70.03 ± 12.90	4.32 ± 0.52	75.33 ± 14.80	6.67 ± 1.09	**0.022**
Week 36 + 1 day	72.52 ± 11.46	2.49 ± 1.08	78.24 ± 15.11	2.91 ± 0.57	**0.010**
Final weight (measured at delivery)	73.06 ± 11.62	0.54 ± 1.02	78.81 ± 15.01	0.57 ± 1.03	**0.009**

**Table 5:** Secondary outcomes in both study groups: IG—intervention group; CG—control group.

	IG (*n* = 69)	CG (*n* = 70)	*p*-Value
	Mean ± SD	Mean ± SD	
Maternal gestational age	39.07 ± 1.37	38.85 ± 1.45	0.363
Birth weight	3197.85 ± 423.95	3187.75 ± 462.37	0.896
Birth length	49.94 ± 2.06	49.57 ± 2.15	0.329
Apgar 1	8.81 ± 0.63	8.71 ± 1.01	0.527
Apgar 5	9.93 ± 0.26	9.80 ± 0.58	0.104
pH cord blood	7.24 ± 0.07	7.23 ± 0.08	0.610
**Mode of delivery**	***n*/%**	***n*/%**	
Non-instrumental	56/81.2	44/62.9	**0.049**
Instrumental	5/7.2	8/11.4	
C-section	8/11.6	10/25.7	
**Type of delivery**	***n*/%**	***n*/%**	
Full term > 37 wk	64/92.8	62/88.6	0.257
Preterm < 37 wk	5/7.2	8/11.4	

## Data Availability

The data are not publicly available due to the agreement between the university and participant hospitals.
